# A differential process mining analysis of COVID-19 management for cancer patients

**DOI:** 10.3389/fonc.2022.1043675

**Published:** 2022-12-07

**Authors:** Michel A. Cuendet, Roberto Gatta, Alexandre Wicky, Camille L. Gerard, Margaux Dalla-Vale, Erica Tavazzi, Grégoire Michielin, Julie Delyon, Julien Cesbron, Sébastien Lofek, Alexandre Huber, Sylvain Pradervand, Olivier Michielin

**Affiliations:** ^1^ Precision Oncology Center, Department of Oncology, Lausanne University Hospital, Lausanne, Switzerland; ^2^ Swiss Institute of Bioinformatics, Lausanne, Switzerland; ^3^ Department of Physiology and Biophysics, Weill Cornell Medicine, New York, NY, United States; ^4^ Department of Clinical and Experimental Sciences, University of Brescia, Brescia, Italy; ^5^ The Francis Crick Institute, London, United Kingdom; ^6^ Department of Information Engineering, University of Padova, Padova, Italy; ^7^ Department of Oncology, Lausanne University Hospital and University of Lausanne, Lausanne, Switzerland

**Keywords:** process mining, COVID-19, oncology, process analysis, clinical pathways

## Abstract

During the acute phase of the COVID-19 pandemic, hospitals faced a challenge to manage patients, especially those with other comorbidities and medical needs, such as cancer patients. Here, we use Process Mining to analyze real-world therapeutic pathways in a cohort of 1182 cancer patients of the Lausanne University Hospital following COVID-19 infection. The algorithm builds trees representing sequences of coarse-grained events such as Home, Hospitalization, Intensive Care and Death. The same trees can also show probability of death or time-to-event statistics in each node. We introduce a new tool, called Differential Process Mining, which enables comparison of two patient strata in each node of the tree, in terms of hits and death rate, together with a statistical significance test. We thus compare management of COVID-19 patients with an active cancer in the first vs. second COVID-19 waves to quantify hospital adaptation to the pandemic. We also compare patients having undergone systemic therapy within 1 year to the rest of the cohort to understand the impact of an active cancer and/or its treatment on COVID-19 outcome. This study demonstrates the value of Process Mining to analyze complex event-based real-world data and generate hypotheses on hospital resource management or on clinical patient care.

## Introduction

Since the end of 2019, the coronavirus disease 2019 (COVID-19) pandemic has caused major medical, social and economic disruption. To date, over 530 million people have been infected and 6.3 million patients have died of the severe acute respiratory syndrome coronavirus 2 (SARS-CoV-2) virus worldwide^1^. From early on during the pandemic, oncological patients have been identified as an at-risk population for the virus (1) with an increased overall risk of death and severe complications compared to patients without cancer (2–5). The outbreak challenged delivery care of past or current cancer patients with delays in screening, diagnosis, follow up, and treatment therapies. Thus, the management of the medical system evolved along the different COVID-19 waves to maintain quality and continuity of cancer care, while mitigating risk of infection transmission.

Neoplastic patients are considered a frail population. They have a higher rate of infection and transmission, likely due to the frequent interaction with the medical environment, and to their immunocompromised state (5–7). In addition, cancer patients have also a higher risk of hospitalization and mortality (8–12). Among this heterogeneous population, multiple risk factors were identified for the SARS-CoV-2 infection. For example, patients with hematologic malignancies and lung cancer have the worse outcomes, which could be explained by the immunosuppressed state of hematological patients and the diminished respiratory capacity of lung cancer patients (3, 10, 13–15).

During the pandemic, hospitals faced unprecedented inpatient admissions as a result of ensuing complications from SARS-CoV-2, which also resulted in workforce restructuring and delays in elective care, including for cancer patients. Therefore, real-time monitoring and retrospective analysis of cancer patient data were essential to investigate the differences in management of cancer patients between the first and the second waves of the pandemic, and to establish whether having recently undergone a systemic cancer treatment impacted COVID-19 outcomes.

Many specific COVID-related clinical endpoints or specific organizational Key Performance Indicators (KPI) have been analysed in the literature (16, 17) but, mostly, they focus on few specific points of the clinical pathway or the organizational workflow (18). More comprehensive approaches able to consider the entire clinical pathways are emerging thanks to a crossover between methods coming from Business Process Analysis and Machine Learning. One of the most representative methods of this kind is Process Mining (PM), which has recently been adapted to healthcare data.

PM is a relatively young discipline (19) aimed at automatically learning which is the most probable process behind a given set of input data called Event Log (EL). A minimal EL is a set of triplets such as {*id*, *date, event*}, where *id* is the patient identifier, *event* is an event that occurred in the patient clinical pathway and *date* is the date corresponding to the event. For each patient, a set of such triplets composes the *trace*, which is the set of events that occurred in their clinical trajectory with the associated timestamps. The most common family of algorithms in PM are meant to perform the so called *Process Discovery* (PD) approach: given an EL as input, the algorithm returns the process, usually in the form of a graph with nodes representing the events and edges the transitions between them. PD is a very generic approach that is commonly applied in many domains, such as manufacturing, services, finance and banking. In the case of healthcare, the specific requirements related to this application domain required the definition of a new perimeter of the discipline called Process Mining for Healthcare (PM4HC) (20). PM4HC adopts the original standpoint of the healthcare domain by taking into account, for example, clinical guidelines (21), or the heterogeneity and the complexity of the data stored in Electronic Health Records (EHR) (22), even if a minimum of data standardisation and quality is needed to warrant the validity of the analysis. Of note, PM/PM4HC are not limited to PD: in the so called *Conformance Checking* (CC) approach, a reference process, such as clinical guidelines, is given together with the empirical patient EL. The algorithm returns a measure of adherence of the process to the data (to measure how the process can represent such data) or of the data to the process (how the patient pathways correctly flow through the reference process; e.g. to measure the adherence to a given clinical guideline). A third approach is called *Process Enhancement* (PE): here a given reference process and empirical EL are analysed together to provide an updated process fitting the input EL better.

PM4HC has been widely used to measure the compliance with clinical guidelines and to discover processes exploiting the data collected in the daily clinical practice, either for clinical or management aims (23, 24). The aforementioned PM modes of action, PD, CC and PE, can be applied towards several goals in healthcare, such as (i) revealing differences in organisation or way to treat among different hospitals; (ii) developing predictive models able to predict the evolution of subsets of patients on the basis of their position in the institutional processes; (iii) developing simulators to estimate the resources required for future steps predicted over a period of time for one or more patients; (iv) identifying bottlenecks in the institutional process with the aim of optimizing value of care.

In particular in oncology, the available literature addresses many of the mentioned endpoints: Binder et al. Huang et al. (25) analyzed skin cancer treatment processes and their compliance with relevant guidelines (26), proposed an original approach to summarize clinical pathways from EL, testing the method on lung, colon and gastric cancers. Lenkowicz et al. (27) measured compliance with clinical guidelines in the treatment of rectal cancer and Tavazzi, Gerard, et al. (28, 29) exploited PD/CC algorithms for representing the clinical pathways of melanoma patients treated with immunotherapy and targeted therapy. Placidi et al. (30) tried to optimize the palliative patient flow in a Radiation Oncology Unit, balancing patient needs and available resources. A practical overview of the most relevant contributions of PM in oncology can be found in Ref (31). PM has also been applied to COVID-19 to show the processes underlying the Intensive Care Unit (ICU) procedures (32), to measure differences in offering the healthcare service in a network of hospitals (33), and to measure the difficulties and the effects of the vaccination strategies in Australia (34).

In this paper, we present a PM analysis aimed at investigating the entire clinical pathways of the cancer patients affected by COVID-19 in our institution. We exploited an approach based on a PD algorithm enriched with tools to support statistical inference in order to highlight remarkable insights in our data. In particular, we present a new method called *differential process mining* (ΔPM) to assess differences between two patient strata in the cohort. With this, we first analysed the differences between the first and the second COVID-19 waves in terms of clinical outcome and institutional assessment. Second, we analyzed the differences in terms of care and mortality between patients who received a systemic treatment for their cancer within the last year versus oncological patients who did not. In the next sections, we introduce the dataset, the tools and the analytical pipeline, and then we present the results for the two PD and ΔPM experiments (first wave vs second wave and recent systemic treatment vs the rest) in terms of descriptive process statistics and of inferential models. Finally, we discuss the results and recapitulate the potential and limitations of this approach.

## Methods

### Data

This study includes all oncological patients followed at the Lausanne University Hospital (CHUV) with a recorded COVID-19 positive test between March 2020 and August 2021. When transforming data from the EHR to an EL amenable to PM, we first aggregated fine-grained information about many consecutive hospitalization events into coarse-grained information with one event per hospital stay. We separated hospitalization events in three categories: regular ward, continuous care, and intensive care, and we discarded outpatient stays. We collected dates of positive COVID-19 tests and subsequent negative tests, as well as death dates. We then discarded events ending more than one day before the first positive COVID-19 test. We also discarded hospitalization events occurring more than 14 days after a positive test or any other contiguous event, the rationale being that if a patient needed to be re-admitted to the hospital for COVID-19 reasons, they would get re-tested. Similarly, we included death events collected from the Swiss Federal Registry of the Persons provided that they occurred within 30 days of a positive COVID-19 test or a contiguous hospitalization event.

### Analysis pipeline

**Figure 1:**
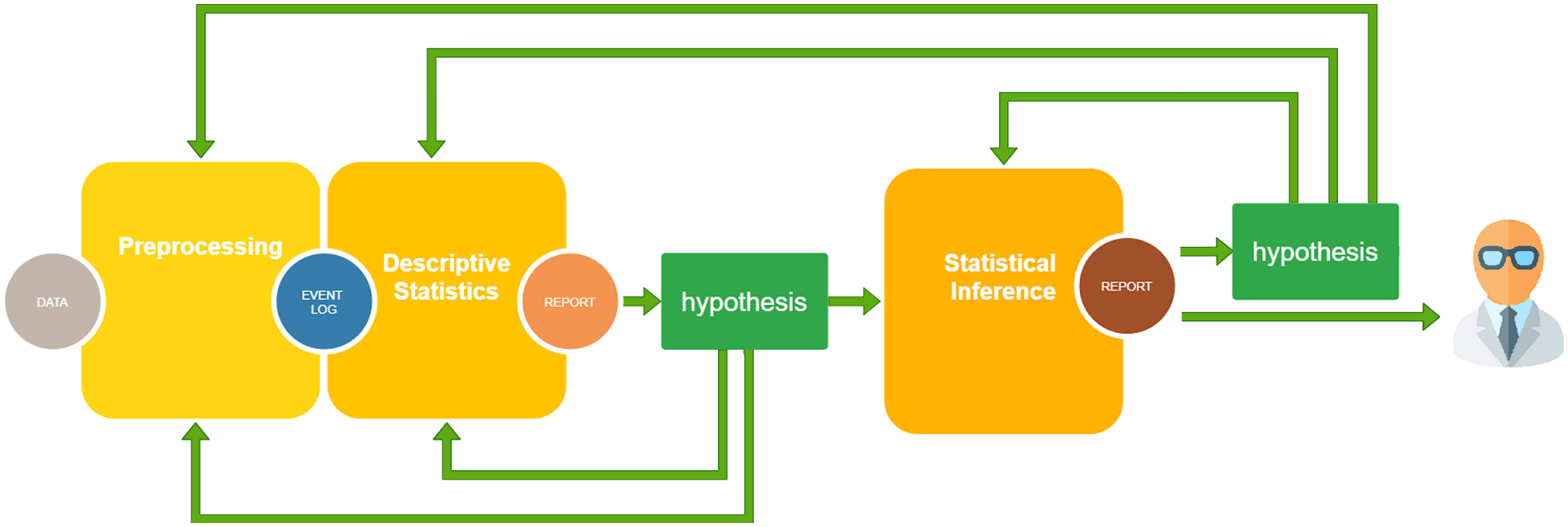
The computational pipeline exploited for the analysis: (i) Preprocessing: data quality is assessed, data with excessive detail are grouped, missing data are removed or imputed and data are shaped in form of an Event Log, the usual input format for Process Mining analysis; (ii) Descriptive Statistics: several indicators are computed on the cohort at hand to suggest some possible hypotheses; (iii) Statistical Inference: statistical significance tests, future event risk predictions, and rationalization of results by domain experts. Here, other hypotheses can be added and the analysis can be enriched, looping back to one of the previous steps.

The computational pipeline followed the iterative paradigm shown in **Figure 1**. Because the processes represent a set of complex time-dependent interactions (e.g. between patients and procedures), the analytical pipeline needs a high level of iteration and interaction with the domain experts to share results, formulate new investigative hypotheses, and loop-back to reprocess the data or fine-tune the analysis (35). This means that with respect to the classical statistical analysis, where the identification of the goal and the methods can be in some way planned at the beginning and pursued with a waterfall approach, the PM pipeline requires a more prototypical approach. The aforementioned pipeline was applied to investigate the two different stratifications within our COVID-19 cohort: (i) the difference between the first and the second COVID-19 waves and (ii) the difference between patients recently under systemic treatment and others, in terms of hits, timing and probabilities.

### Tools

The data were analyzed using Careflow Miner (CFM) (36), a PM algorithm that constructs a tree of the possible clinical pathways. The basic idea of CFM is the following: starting from the root node (here the first positive COVID test), it splits into *n* subsequent nodes representing all possible first events in all traces. Each resulting node contains the number of patients passing through that node and their characteristics. The algorithm is repeated recursively for each new node until we reach the final nodes, when all corresponding traces in the EL are exhausted. Let’s consider, for example, the graph in **Figure 5**. The node on the top means that all the 1182 traces (clinical pathways) begin with a *Start* event. For all traces, the second event is *Covid Test*. Here, the traces admit three possible events as 3rd event: *Home* (67% of traces), *Hospitalisation* (27%) or *Intensive Care* (4%). The sub-cohort passing through *Home* can be split again according to the 4th event present in the corresponding traces, which can be *Death* (21%), *Hospitalisation* (59%) or *Intensive Care* (19%). Here, the missing 1% is due to the traces that stop in this node. To reduce the *Spaghetti Effect* – excessive complexity due to the presence of too many nodes and edges in the graph – the tree can be pruned based either on a threshold on patient number, or on the depth or the tree. The CFM algorithm enables users to build descriptive and inferential trees with different statistics reported in each node: the number of patients passing through the node (*hits*), the median time needed to reach the node from root, and the probability to evolve to a given final outcome such as death or intensive care from that node (prediction).

The adopted software tool was pMineR (37), an R library built for PM4HC. This represents a comfortable development environment for data analysis because it is suitable for statisticians, physicists and physician and therefore increases the amount of potential users. The pMineR library is open-source and is publicly available together with documentation in a GitHub repository^2^. The choice of pMineR was also based on the kind of PD algorithm it proposes: due to the nature of the analysis we focused our attention on CFM because of the simplicity and readability of the produced tree representations. We note that the trees resulting from CFM differ in nature from decision trees, which are supervised machine learning algorithms designed to classify examples (patients) into pre-defined classes based on a number of attributes that can be selected as split criteria at any level of the tree. Instead, CFM trees represent sequences of events whose order strictly follows the data in the input event log. Nonetheless, CFM trees can have a useful predictive value, at least in some nodes, if the final outcome probabilities calculated turn out to show significant variations.

**Figure 2:**
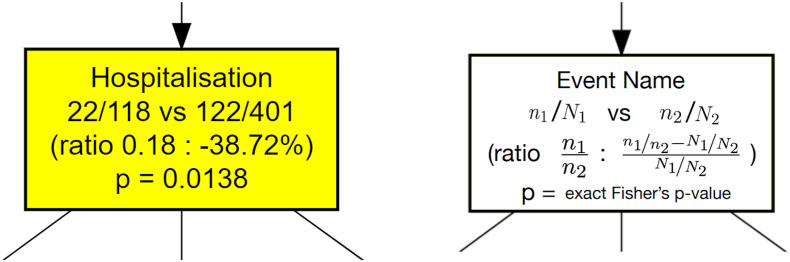
Node contents for a ΔPM analysis of a stratified dataset. An example is shown on the left and the corresponding definitions on the right. The initial dataset is split in two strata with cardinalities *N*_1_ and *N*_2_ . Each node shows the number of hits from both strata, noted *n*_1_ , *n*_2_ , plus the ratio of hits, *n*_1_/*n*_2_ , and the relative change of this ratio compared to the ratio of initial cardinalities, *N*_1_/*N*_2_ . At the bottom, a Fisher’s exact test measures if there is a significant difference between the hit ratio and the original cardinality ratio.

Here, we enriched the CFM algorithm implemented in pMineR with a novel feature that we call *differential process mining* (ΔPM). The aim of ΔPM is to assess the differences between the way two stratified sub-cohorts with *N*_1_ and *N*_2_ patients flow through the same process tree. The tree itself is constructed as in standard CFM using the full cohort. Then, for a given node, assume we have *n*_1_ hits from sub-cohort 1 and *n*_2_ hits from sub-cohort 2. As detailed in **Figure 2**, the ΔPM node will indicate the proportion of hits, *n*_*i*_/*N*_*i*_ , in each sub-cohort *i* , together with the ratio of hits, *n*_1_/*n*_2_ , and the relative change of this ratio compared to the root node, $\frac{n_{1}/n_{2}-N_{1}/N_{2}}{N_{1}/N_{2}}$ . Behind the scene in each node, the following 2x2 contingency table is created, with columns indicating the sub-cohort, and lines whether or not patients passed through the node:

From this table, the equality of the proportions of hits from each sub-cohort can be tested with a Fisher’s exact test. The related p-value indicates the level of statistical significance of the reported proportion difference. Similar measures can be displayed for other node features such as time to reach the node or probability to reach a given event in the future including death rate. Our implementation of ΔPM is available in the current release of the pMineR library^3^. From a well-formatted event log, pMineR functions will automatically generate representations such as **Figures 5**–**9**. An example of generic code to generate a Δ PM tree is given in **Supplementary Figure S1**.

## Results

### Cohort assembly

**Figure 3:**
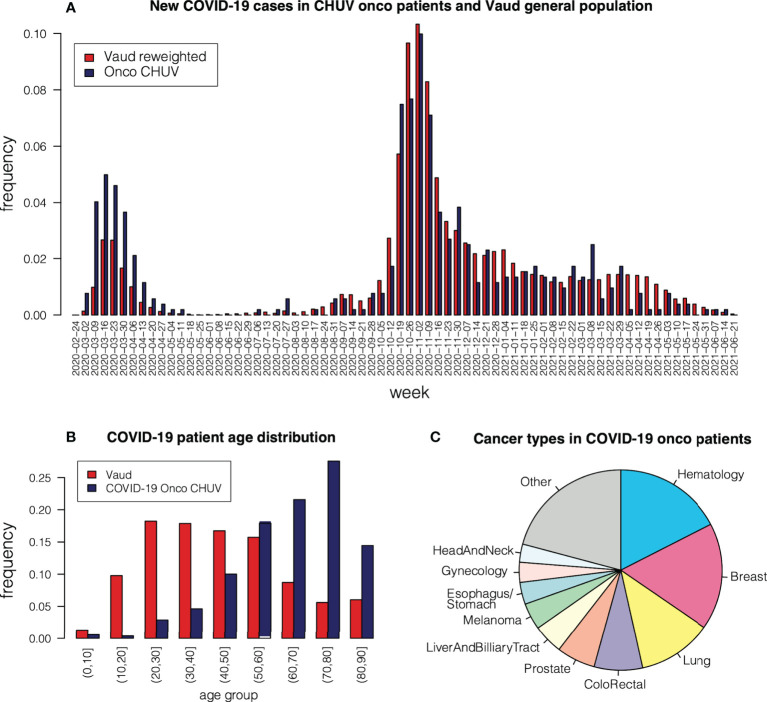
**(A)** Occurrence of COVID-19 cases among CHUV oncological patients, compared to occurrences in the Vaud region re-weighted according to age group. **(B)** Age distribution in oncological COVID-19 patients at CHUV, compared to the general population in the Vaud region. **(C)** Cancer type in COVID-19 patients at CHUV.

For this study, we selected patients followed at the CHUV Oncology Department for a malignant tumor and who presented a positive COVID-19 PCR test. The time distribution of these patients’ first positive COVID-19 tests is shown in **Figure 3A** with label “Onco CHUV”. Two distinct waves are clearly apparent. We assigned patients to the first wave if their first positive test happened in the period between 01.03.2020 and 31.05.2020, and to the second wave between 01.08.2020 and 31.07.2021. After this period there was a third wave, but we decided not to include it in this study as the number of oncological COVID-19 cases had drastically decreased compared to the general population and amounted to <100 patients in total (data not shown). We collected 254 patients in the first wave and 920 patients in the second wave. Adding 8 patients that fell sparsely between waves 1 and 2 and were not assigned to any wave, our cohort is composed of 1182 patients in total, henceforth referred to as the “onco-COVID” patients.

Sub-cohort details are shown on **Table 1**. Among our onco-COVID patients, we identified 524 patients with an *active cancer* as those who had a medical visit related to their cancer in the last 2 years preceding their first positive COVID-19 test. We will use this population to compare clinical journeys between first wave (118 patients) and second wave (401 patients). Further, among our onco-COVID patients, we are going to compare those who have been under systemic treatment (chemotherapy, immunotherapy, hormonotherapy, or targeted therapy) in the last year preceding the COVID-19 test (198 patients) to the rest of the onco-COVID patients (984 patients).

**Table 1:** Patient counts in our onco-COVID cohort.

	Total	Wave 1	Wave 2
All	1182	254	920
Active cancer	524	118	401
Systemic treatment in last year	198	44	101

Red numbers represent the groups used to compare Wave 1 to Wave 2 in active cancer patients. Blue numbers are those used to compare patients recently under systemic treatment (*treatment-active)* to the other oncological patients (1182−198=984 other patients).

**Figure 3B** shows the age distribution of the onco-COVID patients, compared to the general COVID-19-positive population of the Canton of Vaud (Switzerland) in the same period. We observe a strong skew toward older age, due to the concomitant occurrence of cancer in these patients. To compare occurrence of COVID-19 between the onco population and the general population, we re-weighted age groups in the Vaud population so that the age distribution agrees with that of the onco population. We then plotted the re-weighted Vaud weekly occurrences alongside the onco-COVID ones in **Figure 3A**. First, we observe that the first wave hits the onco population more severely than the Vaud population, relative to the second wave. This can be attributed to epidemiological differences between different virus strains, or to more efficient protective measures enacted for sensitive populations during the second wave. Another observation from **Figure 3A** is that the relative proportion of onco patients among COVID-19 infections starts to shrink after March 2021, shortly after the COVID-19 vaccine was introduced in Switzerland. This apparent decrease probably reflects the fact that populations at risk, in particular onco patients, were vaccinated first.

The proportions of cancer types among COVID-19 patients is depicted in **Figure 3C**. The most frequent types are hematological (17%), breast (16%), lung (10%), colo-rectal and anal cancers (9%). Compared to the proportion of hematological cancers in the general population of oncological patients at CHUV (12%), we see an over-representation of COVID-19 occurrence (Chi-square *p*<10^−5^ ), which is consistent with reported trends (3, 10, 13–15) linked to the immunosuppressed state of many patients with hematological malignencies.

### Event log representation

**Figure 4:**
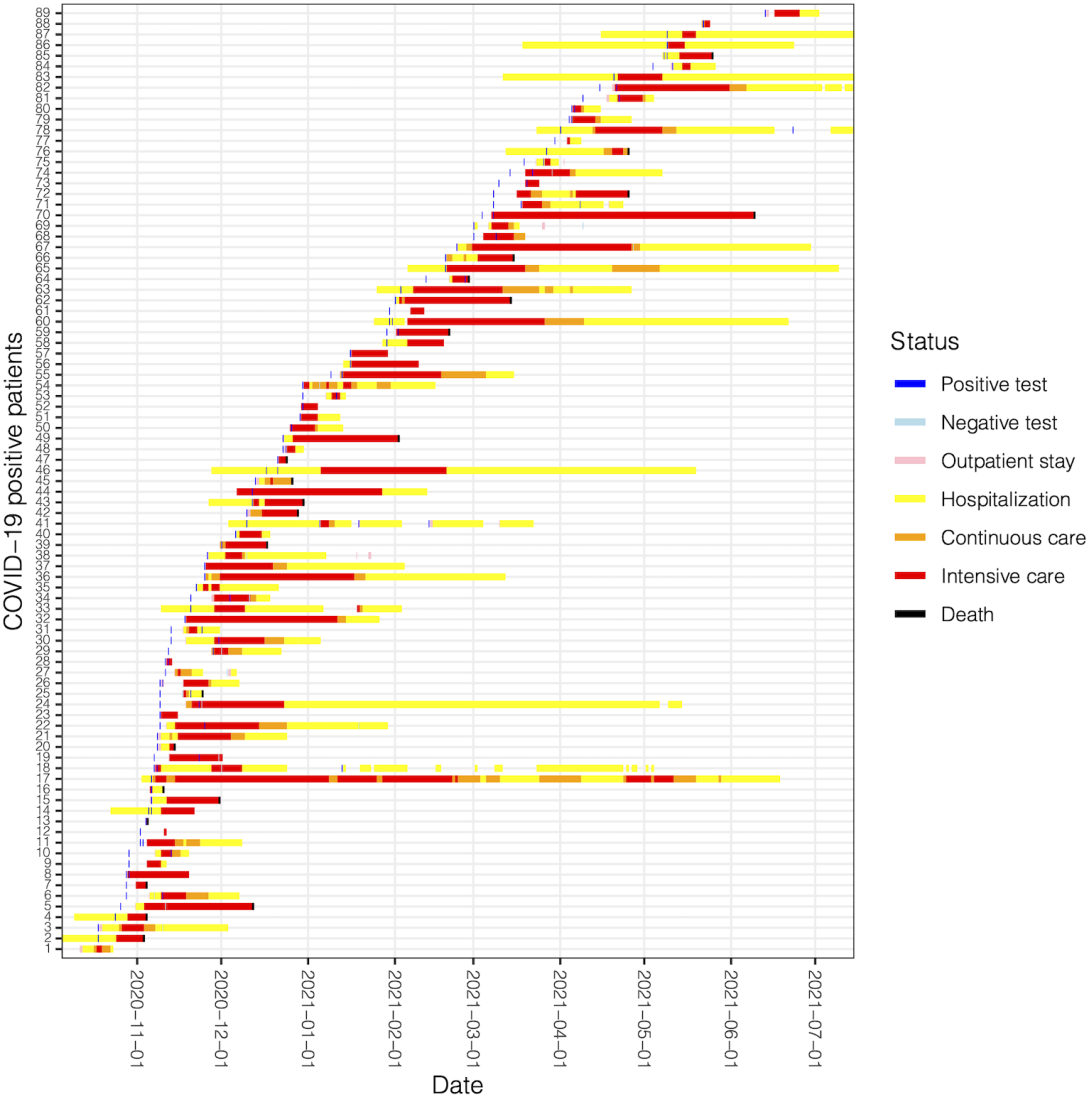
Timeline of patient journeys for active patients of the second wave going through ICU. Each line represents a patient with a coded ID number.

After data pre-processing as described in the Methods Section, we ended up with an EL of 4006 events for our 1182 onco-COVID patients, including COVID-19 tests, regular ward or intensive care hospitalizations, and deaths. Graphical representations are always key to understand a data set and perform essential quality assurance steps. To these ends, we built a timeline representation of curated events on a common time scale for selected groups of patients. An example is shown in **Figure 4** for a specific subset of patients of the second wave that all went through ICU. Patients were sorted by the first test date, so that the apparent slope of event onset reflects the height of the occurrence distribution of **Figure 3A**. We note that in some cases hospitalization extends well beyond the last positive test. In these cases, it cannot be ascertained automatically whether the cause of the hospitalization is COVID-19 or cancer itself or any other cause, thus evidencing an approximation of our study. Of note, over all 1182 onco-COVID patients, only a single one presented a positive COVID-19 test in both the first and the second waves.

### Process analysis

**Figure 5:**
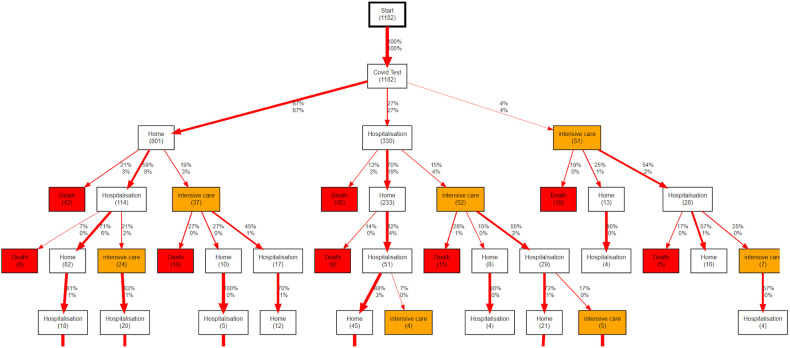
Workflow describing the evolution of COVID-19 patients with a cancer treated at CHUV after the initial *COVID-19 Test*. In each node, the number in parentheses shows the absolute count of patients passing through the node. On each edge, the upper percentage shows the the proportion of patients following that edge relative to the total number of patients exiting the node above (not the number of patients entering that node). The lower percentage shows the proportion of patients following that edge relative to the total number of patients in the root node.

The first useful application of the CFM algorithm is for descriptive analysis of the processes mined. For example, **Figure 5** shows the general workflow for the entire population of the onco-COVID cohort, limited to the sixth level of depth in the discovered tree. After the initial event *COVID-19 test*, four types of events can occur to the patients, corresponding to staying at home (event *Home*), hospitalization in the general ward (event *Hospitalization*), admission to the ICU or continuous care (event *Intensive care*), or event *Death*. Each node shows the number of hits in parentheses. On each edge the two numbers are the percentages of patients flowing through the edge with respect the number of hits in the node above (top), or with respect the entire population (bottom).

The basic descriptive PM analysis in **Figure 5** already allows us to see interesting trends. For example, although about two thirds of the patients remain at home after their initial positive COVID-19 test, 24% of these ((42+114+37)/801) face complications down the line, such as hospitalizations or death. We note that death events directly following a *Home* node include people who may have died at a retirement institution or a palliative care center. Interestingly, regardless of the first choice (*Home, Hospitalization, Intensive care*), we see similar numbers of patients having to go through intensive care further down the path (around 60 patients in each case).

A first enrichment of the basic CFM analysis is the possibility to build a simple but communicative predictor showing in each node the empirical probability to reach a given final endpoint. This tool can be used to estimate how a patient will evolve based on their current position in the tree. We note that the accuracy of this prediction strongly depends on the number of patients involved in the node. Even if one should refrain from using these numbers to predict the fate of single patients, the tool gives an idea about the future clinical evolution of patient groups and about the workload or resource needs for the hospital.

**Figure 6:**
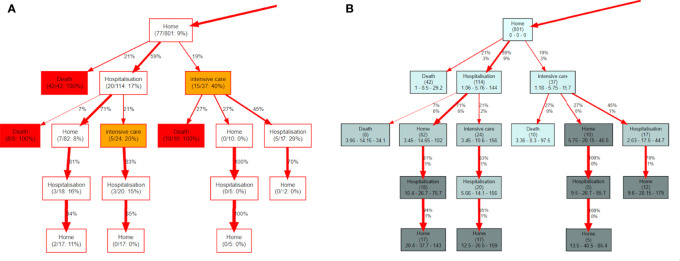
**(A)** represents the left branch of the tree of Figure 5 and shows how the nodes can display information about the probability to die of the patients passing there. **(B)** is built on the same piece of graph and each node contains the time, in days, spent to reach it from the root node. The triplet of numbers represent the minimum, the median and the maximum number of days needed to reach that node.

As a first example of predictive CFM analysis, we focus on death. Using the same graph topology as in **Figure 5**, we can indicate in each node the ratio of patients passing there who are going to die over the total number of hits. We show an example of this approach in **Figure 6A**, which is limited for clarity to the left branch after the *COVID-19 test* event in the whole graph of **Figure 5**. The full tree is available in **Supplementary Figure S2**.

Another interesting variable to observe is the time spent to reach each node. In **Figure 6B** each node displays the number of hits in parentheses and a triplet of numbers indicating the minimum, the median and the maximum time, respectively, needed to reach the node. The full tree is available in **Supplementary Figure S3**.

### Comparison between first and second waves

Here, we applied the ΔPM capability of the CFM implementation in pMineR to compare how two groups of patients flow through the paths and to quantify the significance of the observed differences using statistical tests, as detailed in the Methods section. In the following, we applied ΔPM to two separate comparisons relevant in the context of COVID-19 in onco patients: the difference between first and second COVID-19 waves, and the difference between patients recently under oncological treatment or not. See the Methods Section and **Table 1** for details.

**Figure 7:**
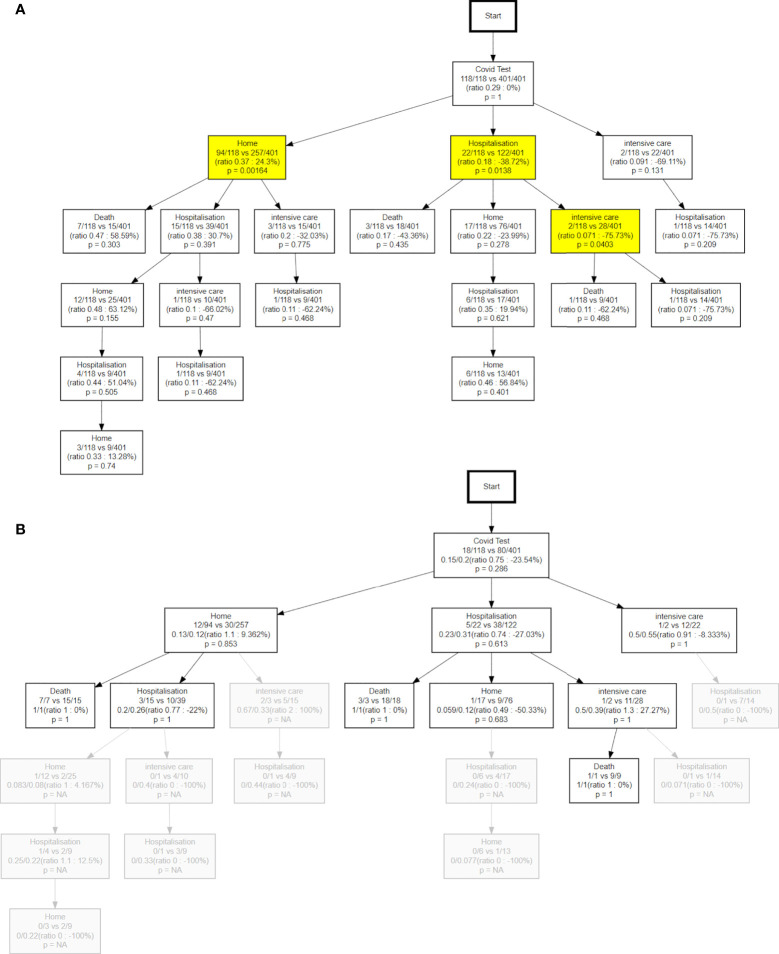
**(A)** differences of hits between the two waves. The percentage value can be interpreted as a higher ratio of wave 1 vs wave 2 relative to the initial ratio at the root of the tree. **(B)** the differences in terms of probability to die between waves. NA, not available.

**Figure 7** shows the ΔPM analysis comparing the first and the second COVID-19 waves, in terms of hits (a) and probability to die (b) for the most representative part of the tree. See the Methods Section and **Figure 2** for details of each node’s contents. The last line contains the p-value of the Fisher’s exact test to check if the difference of hits in waves 1 and 2 is statistically significant. Nodes with a p-value lower than 0.05 are coloured in yellow. In **Figure 7B** each node contains similar information, except that *n*_*i*_ indicates the number of patients who passed through that node in wave *i* that are going to die of COVID-19, and *N*_*i*_ is the total number of hits in the wave *i* . The line below contains the two death rates and the p-value is the Fisher’s exact test to measure if the death rates are statistically different between the two waves.

The most remarkable difference between the two waves concerns the different use of the ICU. The use of this service has more than doubled in the second wave, with respect of the first. **Figure 7A** effectively shows that in the second wave 69% more patients were directed to the ICU right after the initial test. We note that hospitalizations were also on the rise by 39% while a lower proportion of active onco patients could stay at home (-24%). Further, the PM analysis shows an even greater increase (76%) in ICU attendance after the first hospitalization. These trends are also apparent in the time evolution of the different states (see below).

We also observe in **Figure 7B** that the probability to die in the second wave in patients who remained at home after the test (12%) is significantly lower than in patients hospitalized (31%) or sent to ICU (55%). The fact that this difference was not as marked in the first wave (13%, 23%, 50%, respectively), could indicate both a better availability of ICU beds and a more effective patient stratification in the second wave.

### Comparison between patients under systemic treatment or not

**Figure 8:**
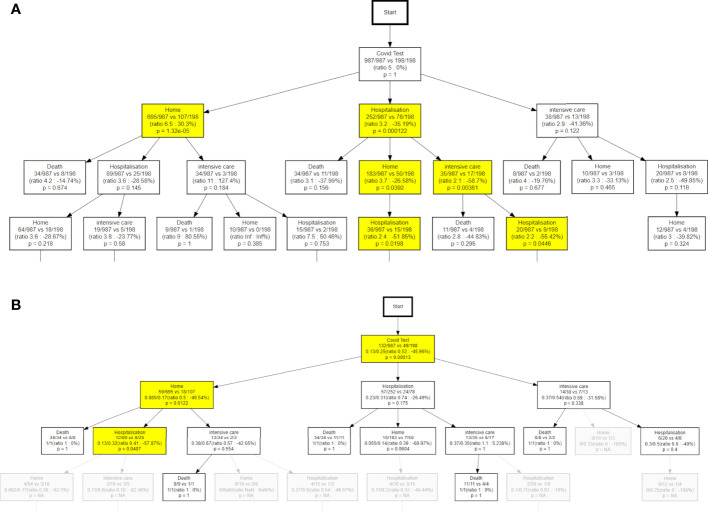
Time evolution of patient number percentages with respect to the total number of patients. **(A)** the solid lines represent the patients in the first wave, and the dotted line the evolution of the patient in the second wave. **(B)** the solid lines show the evolution of patients without recent oncological treatment vs treatment-active patients.

**Figure 8** shows the different paths followed by *treatment-active patients* (those having received a systemic treatment in the year preceding the COVID-19 test) compared to the patients not under active treatment, in terms of hits (a) and probability to die (b). As expected we see in **Figure 8A** that onco patients recently under systemic treatment are around 40% more likely to end up in ICU, 35% more likely to be hospitalized, and 30% less likely to remain at home. Treatment-active patients are even more likely to go to ICU (59% risk increase) after the first hospitalization. This general trend could be due to two factors: either the patients were more impacted by the COVID-19 infection, or the physicians decided to minimize risk considering cancer as a comorbidity and prescribed preventive hospitalizations, or both. We note that many nodes below the first *Hospitalization* node show the same trend of increased risk for treatment-active patients. This is expected as imbalances propagate downstream of the node where they first originated.

Differences are also evident for the death rates. **Figure 8B** shows a higher overall probability to die in the treatment-active patients compared to the rest of the cohort (25% vs 13%). Despite a higher intensity of care for these patients as noted above, we still see a higher death rate among patients remaining at home after the test (**Figure 8B**, 17% vs 8%). The difference is even more pronounced for patients who get re-hospitalized (32% vs 13%). Note that COVID-related death rates might be slightly overestimated in the active onco patients, due to some deaths related to cancer being more likely to happen in the relevant time interval.

**Figure 9:**
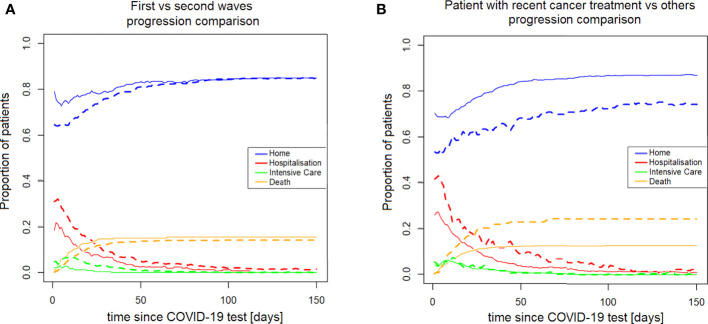
Both **(A, B)** refer to the comparison of the treatment-active patients to the other oncopatients; **(A)** show the differences in terms of hits **(B)** the different probability to die. NA, not available.

From a clinical monitoring perspective, it is useful to have a synthetic vision of how many patients are in each specific state as a function time. This allows, for example, monitoring the pressure on the ICU at different phases of the pandemic. **Figure 9** shows how the percentages of patients among the different possible states (*Home*, *Hospitalization*, *Intensive care*, *Death*) evolve over time, comparing the first vs second wave, and patients who recently received systemic treatments vs those who did not.

## Discussion

In this study, we used the innovative PM approach to gain interesting insights on the pathways of our onco patients at CHUV after a positive COVID-19 test, through the categorized steps of *Home, Hospitalization, Intensive care*, and *Death*. In particular, we introduced a new analysis modality that we called ΔPM, which allows researchers to quantify differences between two patient strata in terms of hits, or in terms of occurrence of a final outcome such as death. We used the ΔPM approach to compare the first and second COVID-19 waves in onco-active patients, and to compare patients recently under systemic treatment to the rest of the onco-COVID cohort. The results we presented are interesting from three different points of view. First, this analysis suggested some hypotheses on COVID-19 management in onco patients, which deserve to be investigated further in more specific studies. The second perspective on our PM analysis regards the organizational response to the epidemic emergency, between the first and the second wave, for example in measuring the dramatic changes in the patient’s pathways. Third, innovative methodology to analyse the data, such as ΔPM, is of interest for Data Analysts who will be able to readily apply it to their own datasets using the publicly-available pMineR implementation.

We note that drawing definitive conclusions about the clinical and organizational aspects is hindered by many confounding factors. For example, the clinical knowledge acquired between the waves, the adaptation of patient behaviours, the difference in epidemiological exposure between the first and the second waves, and the differences in virus strains make a direct comparison difficult. While the first wave was an unplanned response to a particularly stressful and unknown challenge, the second wave raised when the hospital had had time to organize their resources and services for an appropriate response. Similarly, in the second wave, COVID-19 patient care benefited from a better synergy among the hospitals in the area, planning different levels of care depending on the available infrastructure. This led to a better selection of patients admitted in the second wave. Nevertheless, looking at the results taken together, some general trends could be clearly identified and some hypotheses could be formulated.

From the institutional perspective, the expectation of hospitalisation, intensive care, home assistance, and death, are shown in **Figure 9**: according to the aforementioned discussion, the institutional behaviour, in the second wave, was more oriented toward hospitalization, even if this did not mean a significant reduction of mortality. This point is counter-intuitive but might be explained by different reasons. For example a general strategy was put in place in the State of Vaud to send more serious patients to CHUV in the second wave (as opposed to treating them at home or in regional centers). Or this trend may reflect an unsuccessful attempt to reduce the mortality, or just the greater availability of hospital beds in the second wave, when the hospital was prepared. Interestingly, **Figure 9A** shows that the difference in institutional approach between the first and second waves was most pronounced in the first days after the positive COVID-19 test, and tended to vanish later on. Notably, the mortality almost overlapped during all the 0-150 days of observation. In **Figure 9B**, the treatment-active patients show a constant higher need of clinical assistance and a higher probability to die in comparison of the other patients. This point is quite expected, due to the higher frailty of patients recently under systemic treatment that could be explained by the activity of their cancer and the stress of therapies.

## Conclusion

In this paper we analysed a set of oncological patients affected by COVID-19 to address two different goals: to investigate the differences between the first and the second wave, and to analyse the difference in mortality and hospital care between patients under active systemic treatment and others. To do that, we exploited a modern discipline called Process Mining, which analyzes the data considering the different clinical pathways of the observed patients and displays the results in few, communicative graphs. All observations of the Results Section were made possible by this process-oriented approach to data analysis. The CFM algorithms, in particular, provide a useful way to quickly obtain a comprehensive but intuitive data representation. We further enriched the standard PM algorithms with our new ΔPM tools that combine the benefits of the Process Discovery approach with the more usual statistical tools in order to analyse stratified cohorts and assess differences in terms of hits, time to node, or mortality. The implementation of inferential statistical tests enriches the readability and makes this tool suitable to suggest further hypotheses. While similar considerations can be made with more common statistical tools once a specific question is posed, the possibility to observe all branches of the tree simultaneously allows us to explore and direct downstream analysis where the data seems to have the most to reveal.

This work confirmed that PM is an effective tool to tackle complex event-based datasets containing a large number of realizations of an underlying process. By laying bare the data in its full temporal complexity, This approach can reveal specific insights which can remain hidden with a more general statistical analysis. We think that this is probably the most remarkable point: while PM is often used to test hypotheses, in our case we found that mining the data with PM tools turned out to be an efficient way of generating new hypotheses. Indeed, PM brought about new questions more than new answers. This might seem a limitation but we think otherwise: in our case, for example, the similar mortality between the two waves is suggesting that something happened in terms of protocols or recruitment or quality of cares (or, maybe, all of them). Some more details of the admitted patients or more bio-metric measures could allow us to understand in which part of the clinical pathways the performances dropped. However, the limited number of patients and the limited set of covariates available automatically in the EHR are clear limitations of this analysis.

More in general, looking back to this COVID-19 epidemics, it was a challenge for real-world data analysis due to several reasons: (i) the medical knowledge evolved quickly, with often unclear and shifting standards of care, and this should be taken into account when comparing cohorts acquired at different periods of time. (ii) in addition, hospital policies changed from one wave to the next, for example on resource management, which might have impacts at many levels. Last but not least, (iii) COVID-19 was not an equal challenge for all hospitals, as it was a challenge for the entire healthcare system. For example, patients were admitted depending on the available regional resources (for example, at a certain point the healthcare authorities tried to keep some hospitals COVID-free, to be able to offer services to the non-COVID patients). Even with the many limitations outlined above, this study demonstrated the usefulness of PM to quickly analyse data and improve our capabilities to cope with the future expected or unexpected clinical challenges.

## Data availability statement

The raw data supporting the conclusions of this article will be made available by the authors, without undue reservation, upon approval by the relevant Ethics Committee.

## Ethics statement

This study qualifies as a quality control study according to the Swiss Human Research Act, in which case usage of retrospective patient data is not subjected to ethics approval.We did not include data of patients who explicitly refused the CHUV general consent for health-related data re-use.

## Author contributions

Clinical data collection: AW, MD-V, CG, GM, JD, JC, SL, AH. Methodological developments: MC, RG, AW, OM. Data analysis: MC, RG, AW, MD-V, CG, ET, SP, OM. Manuscript writing: MC, RG, CG. All authors reviewed the article and approved the submitted version.

## Funding

Open access funding was provided by the University of Lausanne.

## Acknowledgments

We acknowledge the medical teams that took care of the patients during the pandemic, in particular members of the Medical Oncology Division of CHUV.

## Conflict of interest

The authors declare that the research was conducted in the absence of any commercial or financial relationships that could be construed as a potential conflict of interest.

## Correction note

A correction has been made to this article. Details can be found at: 10.3389/fonc.2026.1777196.

## Publisher’s note

All claims expressed in this article are solely those of the authors and do not necessarily represent those of their affiliated organizations, or those of the publisher, the editors and the reviewers. Any product that may be evaluated in this article, or claim that may be made by its manufacturer, is not guaranteed or endorsed by the publisher.

## Footnotes

1
https://covid19.who.int/ (28th of June 2022)

2
https://github.com/PMLiquidLab/pMineR.v046

3
https://github.com/PMLiquidLab/pMineR.v046
