# Erratum: A differential process mining analysis of COVID-19 management for cancer patients

**DOI:** 10.3389/fonc.2023.1173233

**Published:** 2023-03-08

**Authors:** Frontiers Production Office

**Affiliations:** Frontiers Media SA, Lausanne, Switzerland

**Keywords:** process mining, COVID-19, oncology, process analysis, clinical pathways

An omission to the funding section of the original article was made in error. The following sentence has been added: “Open access funding was provided by the University of Lausanne”.

The original version of this article has been updated.

