# Correction: A differential process mining analysis of COVID-19 management for cancer patients

**DOI:** 10.3389/fonc.2026.1777196

**Published:** 2026-03-02

**Authors:** Michel A. Cuendet, Roberto Gatta, Alexandre Wicky, Camille L. Gerard, Margaux Dalla-Vale, Erica Tavazzi, Grégoire Michielin, Julie Delyon, Julien Cesbron, Sébastien Lofek, Alexandre Huber, Sylvain Pradervand, Olivier Michielin

**Affiliations:** 1 Precision Oncology Center, Department of Oncology, Lausanne University Hospital, Lausanne, Switzerland; 2 Swiss Institute of Bioinformatics, Lausanne, Switzerland; 3 Department of Physiology and Biophysics, Weill Cornell Medicine, New York, NY, United States; 4 Department of Clinical and Experimental Sciences, University of Brescia, Brescia, Italy; 5 The Francis Crick Institute, London, United Kingdom; 6 Department of Information Engineering, University of Padova, Padova, Italy; 7 Department of Oncology, Lausanne University Hospital and University of Lausanne, Lausanne, Switzerland

**Keywords:** process mining, COVID-19, oncology, process analysis, clinical pathways

Eight individuals listed as authors in the published article do not qualify for authorship and are no longer included. These are: Nabila Ferahta, Jeremy Jankovic, Rita Demicheli, Hasna Bouchaab, Antonia Digklia, Michel Obeid, Solange Peters, Manuela Eicher. The correct author list reads:

*“Cuendet MA, Gatta R, Wicky A, Gerard CL, Dalla-Vale M, Tavazzi E, Michielin G, Delyon J, Cesbron J, Lofek S, Huber A, Pradervand S and Michielin O.”*

The **Author Contributions** Statement has been corrected to read:

“Clinical data collection: AW, MD-V, CG, GM, JD, JC, SL, AH. Methodological developments: MC, RG, AW, OM. Data analysis: MC, RG, AW, MD-V, CG, ET, SP, OM. Manuscript writing: MC, RG, CG”. All authors reviewed the article and approved the submitted version.

Following the withdrawal of authors, a correction has been made to the **Acknowledgements**.

“We acknowledge the medical teams that took care of the patients during the pandemic, in particular members of the Medical Oncology Division of CHUV.”

The **Ethics statement** was erroneously given as “The usage of retrospective patient data for this study was approved by the local Ethics Committee — Vaud Canton, Switzerland (Protocol No. 2019-00448). The patients/participants provided their written informed consent to participate in this study.” However, the reference to this protocol was unnecessary. The correct ethics statement is:

**Ethics Statement**

This study qualifies as a quality control study according to the Swiss Human Research Act, in which case usage of retrospective patient data is not subjected to ethics approval. We did not include data of patients who explicitly refused the CHUV general consent for health-related data re-use.

The original version of this article has been updated.

