# Supplementary material for: A differential process mining analysis of COVID-19 management for cancer patients
Source: Front Oncol. 2022 Dec 7;12:1043675. doi: 10.3389/fonc.2022.1043675 (PMC9768429; doi:10.3389/fonc.2022.1043675)
Supplement: Supplementary file 1 [file DataSheet_1.pdf]

frontiers

Supplementary Material

1 SUPPLEMENTARY FIGURES

```r
# Load the data.
# The original event log (dataframe event_log) should have columns with the following columns:
#   - PATIENT_ID: unique patient identification number
#   - EVENT_TYPE: one of a few pre-defined event types (Home, Hospitalization, Instensive Care, Death, Home)
#   - START_DATE: start date for the event with format Y-m-d H:M:S
#   - STRATUM: The patient group, with values 1 or 2

tmp.objDL <- dataLoader(verbose.mode = FALSE)
tmp.objDL$load.data.frame( mydata = data.frame(event_log) ,
            IDName = "PATIENT_ID",
            EVENTName = "EVENT_TYPE",
            dateColumnName = "START_DATE",
            format.column.date = "%Y-%m-%d %H:%M:%S" ))
formatted_data <- tmp.objDL$getData()

# Build CareFlowMiner object
cfm_object <- careFlowMiner()
cfm_object$loadDataset(inputData = formatted_data)
cfm_tree <- cfm_object$plotCFGraphComparison(stratifyFor = "STRATUM",
            fisher.threshold = 0.05,
            stratificationValues = c("1","2"),
            checkDurationFromRoot = FALSE,
            finalStateForHits = c("Death"),
            hitsMeansReachAGivenFinalState = TRUE,
            nodeShape = "box",
            kindOfGraph = "dot",
            show.far.leaf = FALSE,
            depth = Inf,
            abs.threshold = 10)

# Plot the tree
grViz(cfm_tree$script)
```

Figure S1. Generic R code snippet showing how functions form the pMineR library can be used to generate a ∆PM tree starting from a data frame containing the event log. Details are given in the code comments.

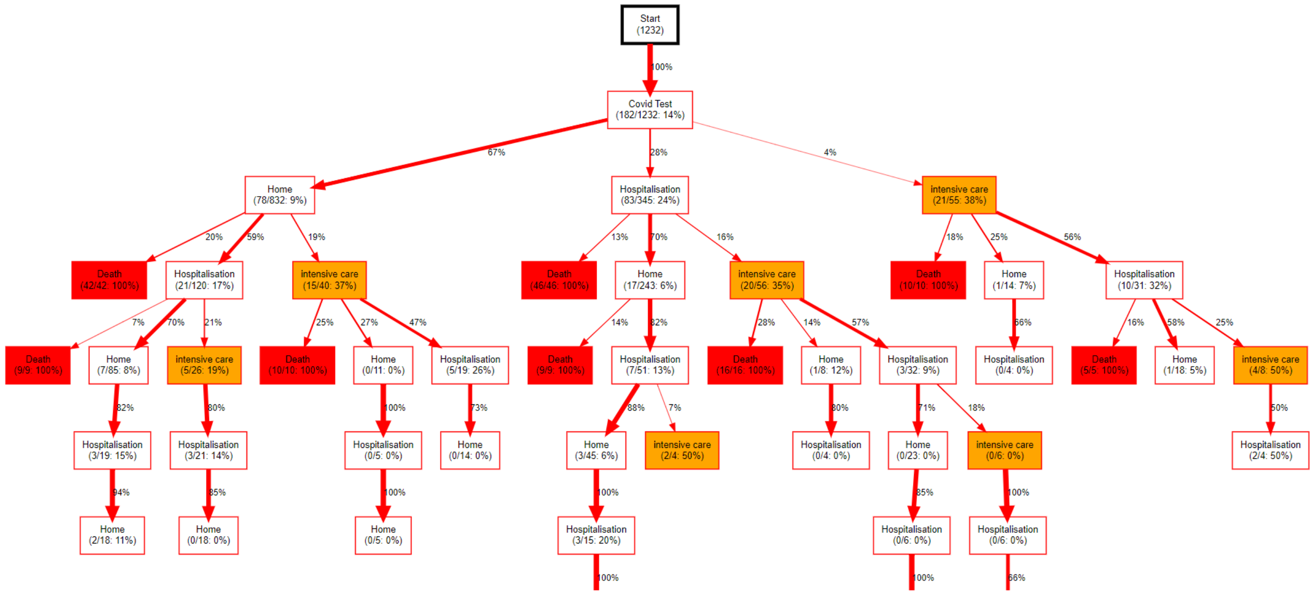

Figure S2. Full process tree for the onco-COVID cohort with the probability to die reported in each node.

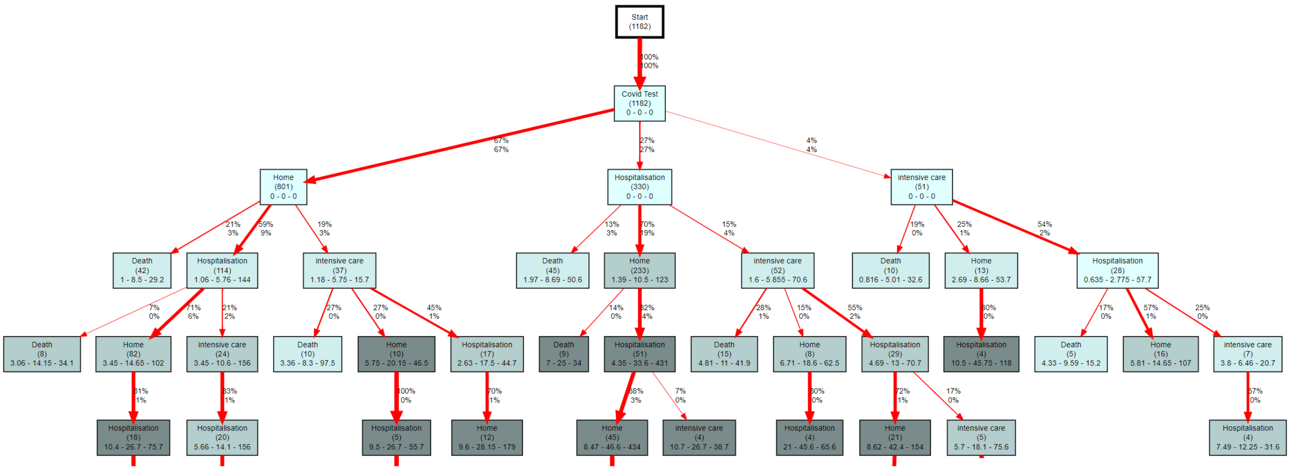

Figure S3. Full process tree for the onco-COVID cohort with the time to event reported in each node. The triplet on the lower line indicated the minimum, median and maximum time in days since the COVID-19 test.
